# Autonomous Robotic Point-of-Care Ultrasound Imaging for Monitoring of COVID-19–Induced Pulmonary Diseases

**DOI:** 10.3389/frobt.2021.645756

**Published:** 2021-05-25

**Authors:** Lidia Al-Zogbi, Vivek Singh, Brian Teixeira, Avani Ahuja, Pooyan Sahbaee Bagherzadeh, Ankur Kapoor, Hamed Saeidi, Thorsten Fleiter, Axel Krieger

**Affiliations:** ^1^ Laboratory for Computational Sensing and Robotics, Department of Mechanical Engineering, Johns Hopkins University, Baltimore, MD, United States; ^2^ Medical Imaging Technologies, Siemens Medical Solutions, Inc. USA, Princeton, NJ, United States; ^3^ Georgetown Day High School, WA, DC, United States; ^4^ R. Cowley Shock Trauma Center, Department of Diagnostic Radiology, School of Medicine, University of Maryland, Baltimore, MD, United States

**Keywords:** autonomous robotics, point-of-care ultrasound, force feedback, 3D landmark estimation, 3D deep convolutional network, COVID-19

## Abstract

The COVID-19 pandemic has emerged as a serious global health crisis, with the predominant morbidity and mortality linked to pulmonary involvement. Point-of-Care ultrasound (POCUS) scanning, becoming one of the primary determinative methods for its diagnosis and staging, requires, however, close contact of healthcare workers with patients, therefore increasing the risk of infection. This work thus proposes an autonomous robotic solution that enables POCUS scanning of COVID-19 patients’ lungs for diagnosis and staging. An algorithm was developed for approximating the optimal position of an ultrasound probe on a patient from prior CT scans to reach predefined lung infiltrates. In the absence of prior CT scans, a deep learning method was developed for predicting 3D landmark positions of a human ribcage given a torso surface model. The landmarks, combined with the surface model, are subsequently used for estimating optimal ultrasound probe position on the patient for imaging infiltrates. These algorithms, combined with a force–displacement profile collection methodology, enabled the system to successfully image all points of interest in a simulated experimental setup with an average accuracy of 20.6 ± 14.7 mm using prior CT scans, and 19.8 ± 16.9 mm using only ribcage landmark estimation. A study on a full torso ultrasound phantom showed that autonomously acquired ultrasound images were 100% interpretable when using force feedback with prior CT and 88% with landmark estimation, compared to 75 and 58% without force feedback, respectively. This demonstrates the preliminary feasibility of the system, and its potential for offering a solution to help mitigate the spread of COVID-19 in vulnerable environments.

## 1 Introduction

The COVID-19 pandemic has emerged as a serious global health crisis, with the primary morbidity and mortality linked to pulmonary involvement. Prompt and accurate diagnostic assessment is thus crucial for understanding and controlling the spread of the disease, with Point-of-Care Ultrasound scanning (POCUS) becoming one of the primary determinative methods for its diagnosis and staging (Buda et al., 2020). Although safer and more efficient than other imaging modalities (Peng et al., 2020), POCUS requires close contact of radiologists and ultrasound technicians with patients, subsequently increasing risk for infections (Abramowicz and Basseal, 2020). To that end, robotic solutions embody an opportunity to offer a safer and more efficient environment to help mitigate the critical need for monitoring patients’ lungs in the COVID-19 pandemic, as well as other infectious diseases.

Tele-operated solutions allow medical experts to remotely control the positioning of an ultrasound (US) probe attached to a robotic system, thus reducing the distance between medical personnel and patients to a safer margin. Several tele-operated systems have been successfully tested amid the pandemic for various purposes. Ye et al. (2020) developed a 5G-based robot-assisted remote US system for the assessment of the heart and lungs of COVID-19 patients, whereby the system successfully evaluated lung lesions and pericardial effusions in patients with varying levels of disease progression. An MGIUS-R3 tele-echography system was also evaluated for remote diagnosis of pneumonia in COVID-19 patients (Wu et al., 2020). The physician successfully obtained a lung scan from 700 km away from the patient’s site, allowing him to diagnose lung pneumonia characterized by pleural abnormalities. The system could also detect left ventricular systolic function, as well as other complications such as venous thrombosis (Wang et al., 2020). Yang et al. (2020) developed a tele-operated system that, in addition to performing robotized US, is capable of medicine delivery, operation of medical instruments, and extensive disinfection of high-touch surfaces.

Although a better alternative to traditional in-person POCUS, commercialized tele-operated solutions nonetheless typically involve the presence of at least one healthcare worker in close vicinity of the patient to initialize the setup and assist the remote sonographer (Adams et al., 2020). An autonomous robotic US solution would hence further limit the required physical interaction between healthcare workers and infected patients, while offering more accuracy and repeatability to enhance imaging results, and hence patient outcomes. An autonomous solution can additionally become a valuable tool for assisting less experienced healthcare workers, especially amid the COVID-19 pandemic where trained medical personnel is such a scarce resource. Mylonas et al. (2013) have trained a KUKA light-weight robotic arm using learning-from-demonstration to conduct autonomous US scans; however, the entire setup was trained and evaluated on Latin letters detection within a uniform and clearly defined workspace, which is not easily transferable to applications involving human anatomy. An autonomous US robotic solution has been developed by Virga et al. (2016) for scanning abdominal aortic aneurysms, whereby the optimal probe pressure is estimated offline for enhanced image acquisition, and the probe’s orientation estimated online for maximizing the aorta’s visibility. Kim et al. (2017) designed a control algorithm for an US scanning robot using US images and force measurements as feedback tested on a thyroid phantom. The authors developed their own robotic manipulator, which, however, has too limited of a reach to scan a human upper torso. Additionally, the phantom on which the experiments were conducted is flat without an embedded skeleton, impeding the solution’s immediate translation to lung scanning. The aforementioned works show significant progress in the field; however, to the best of our knowledge, no system has been developed for performing US lung scans in particular. Robotic POCUS of lungs requires a more tailored approach due to 1) the large volume of the organ which cannot be inspected in a single US scan, implying that during each session, multiple scans from different locations need to be sequentially collected for monitoring the disease’s progression; 2) the scattering of US rays through lung air, meaning that an autonomous solution needs to be patient-specific to account for different lung shapes and sizes to minimize this effect; and finally 3) the potential obstruction of the lungs by the ribcage, which would result in an uninterpretable scan.

We thus developed and implemented a protocol for performing autonomous robotic POCUS scanning on COVID-19 patients’ lungs for diagnosis and monitoring purposes. The major contributions of our work are 1) improved lung US scans using solely force feedback, given a patient’s prior CT scan; and 2) prediction of anatomical features of a patient’s ribcage using only a surface torso model to eliminate the need for a CT scan, with comparable results in terms of US scan accuracy and quality. This article is organized as follows: 1) Materials and Methods, which includes detailed information on the adopted robotic scanning procedure and algorithmic steps at every stage of the protocol; 2) Experiments and Results, which details the experimental setup used for validating the approach as well as the obtained results; 3) Discussion, providing an insightful analysis of aforementioned results and future work prospects; and finally 4) Conclusions, summarizing our key contributions and research outcomes.

## 2 Materials and Methods

### 2.1 Testbed

**FIGURE 1:**
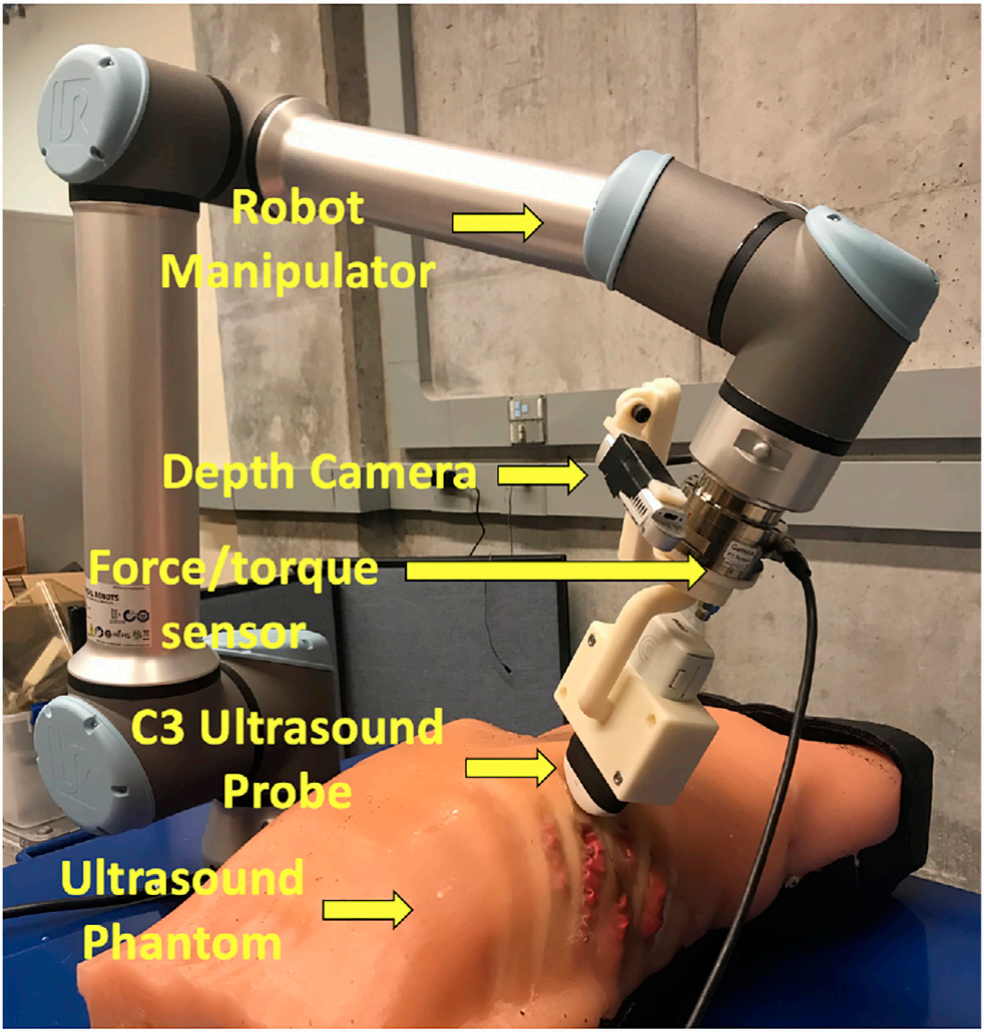
Components of the autonomous robotic system setup.

The overall robotic setup is shown in Figure 1, which consists of a 6 degrees of freedom UR10e robot (Universal Robot, Odense, Denmark), a world frame color camera Intel RealSense D415 (Intel, Santa Clara, California, United States), a C3 wireless US probe (Clarius, Burnaby, British Columbia, Canada), and an SI-65-5 six-axis F/T Gamma transducer (ATI Industrial, Apex, North Carolina, United States). The US probe and camera are attached to the robot’s end effector in series with the force sensor via a custom-designed 3D-printed mount for measuring the forces along the US probe’s tip. The tool camera was positioned behind the probe to visualize it in the camera frame, as well as the scene in front of it. An M15 Alienware laptop (Dell, Round Rock, Texas, United States) with a single 6 GB NVIDIA GeForce GTX 1660 Ti GPU memory card was used for controlling the robot.

For the experimental validation of the results, we are using a custom-made full torso patient-specific ultrasound phantom that was originally developed for FAST scan evaluation, as it offers a realistic model of a patient’s torso (Al-Zogbi et al., 2020). The phantom’s geometry and organs were obtained from the CT scan of a 32-year-old anonymized male patient with low body mass index (BMI). The tissues were made of a combination of two different ballistic gelatin materials to achieve human-like stiffness, cast into the 3D-printed molds derived from the CT scan. The skeleton was 3D printed in polycarbonate, and the mechanical and acoustic properties of the phantom were evaluated, showing great similitude to human tissue properties. An expert radiologist has also positively reviewed the phantom under US imaging.

### 2.2 Workflow

**FIGURE 2:**
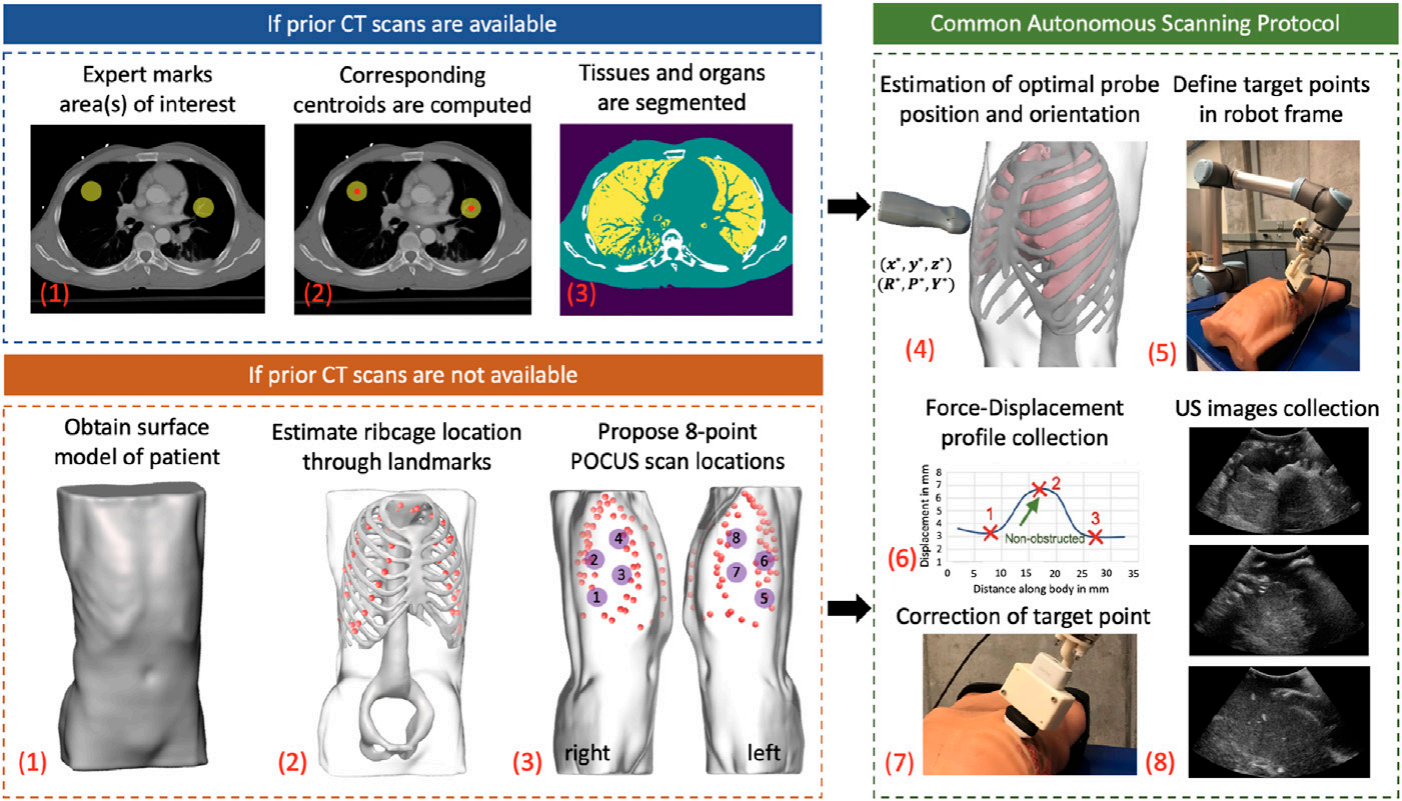
General workflow of the autonomous robotic setup. Areas of interest in lungs are marked by an expert if a CT scan of a patient is available. The centroid of each area is computed, and an algorithm segments different organs in CT images. In the case where a CT is not available, a surface model of the patient is obtained using a depth camera, which acts as an input to a deep learning algorithm to estimate ribcage landmarks. The 8-point POCUS locations are subsequently manually proposed. In both cases, the optimal US probe positioning and orientation is estimated, which is relayed to the robotic system. During implementation, a force–displacement profile is collected, which is used to correct the end effector’s position to avoid imaging of bones. US images of the target point are finally collected in a sweeping motion of $\pm 30^{\circ }$.

Figure 2 shows the step-by-step workflow of the autonomous US scanning protocol of the robotic system, with and without prior CT scans. First, an expert radiologist marks on a chest CT of a patient regions of interest (typically but not necessarily containing infiltrates), which are to be observed over the course of coming days to evaluate the progression of the disease. An algorithm computes the spatial centroid of each region and returns optimal positions and orientations of an US probe on a subject’s body, such that the resultant US image guarantees to contain the specified point of interest without skeletal obstruction. In the case where a CT scan is not available, landmarks of the ribcage are estimated using the patient’s 3D mesh model, which is obtained through a depth camera. The scanning points are then manually selected on the model following the 8-point POCUS protocol (Blaivas, 2012). Goal positions and orientations are then relayed to the robotic system. Owing to possible kinematic and registration errors, the positioning of the US probe may suffer from unknown displacements, which can compromise the quality and thus interpretability of the US scans. To mitigate this, the system employs a force–feedback mechanism through the US probe to avoid skeletal structures that will lead to shadowing. The specifics of this protocol are discussed in further detail in the following subsections.

The optimal US scanning position and orientation algorithm, as well as the ribcage landmark estimation, were implemented in Python, whereas the robot control, which includes planning and data processing algorithms, was integrated via Robot Operating System (Quigley et al., 2009). Kinematics and Dynamics Library (KDL) in Open Robot Control Systems (OROCOS) (Smits et al., 2011) is used to transform the task-space trajectories of the robot to the joint space trajectories, which are the final output of the high-level autonomous control system. The drivers developed by Universal Robot allow one to apply the low-level controllers to the robot to follow the desired joint-space trajectories.

### 2.3 Estimation of Optimal Ultrasound Probe Positioning and Orientation

**FIGURE 3:**
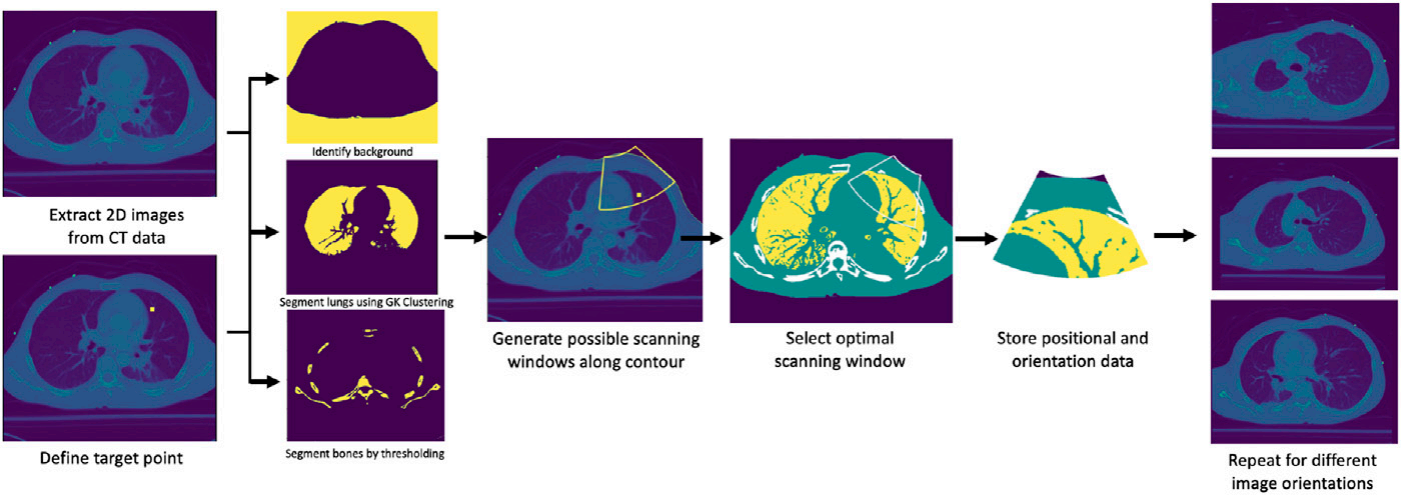
Overview of steps for optimal scanning points selection algorithm.

A prior CT scan of a patient’s chest is required for the algorithm’s deployment. The possible scanning area on the body of a given patient is limited to the frontal and side regions. Given a region of interest in the lungs specified by a medical expert, its spatial centroid is computed first to define a target point. The procedure thus initially targets imaging a single point, followed by a sweeping motion about the contact line of the US probe to encompass the surrounding area. A set of images from the CT data containing the computed centroid is generated at various orientations, each of which is subsequently segmented into four major classes: 1) soft tissues, 2) lung air, 3) bones, and 4) background. First, the background is identified using a combination of thresholding, morphological transformations, and spacial context information. The inverse of the resultant binary mask thus delimits the patient’s anatomy from the background. The inverse mask is next used to restrict the region in which lung air is identified and segmented through Gustafson–Kessel clustering (Elsayad, 2008). The bones are segmented using an intensity thresholding cutoff of 1,250, combined with spacial context information. Soft tissues are lastly identified by subtracting the bones and lung air masks from the inverse of the background mask. For simplicity’s sake, identical acoustic properties are considered for all soft tissues within the patient’s body. This assumption is justified by comparing the acoustic properties of various organs in the lungs’ vicinity (soft tissue, liver, and spleen), which were shown to be sufficiently close to each other. Figure 3 summarizes the workflow of the segmentation algorithms, as well as the final segmentation masks.

The objective is to image predetermined points within the lungs, while maximizing the quality of the US scan which is influenced by three major factors: 1) proximity of the target to the US probe, 2) medium through which the US beam travels, and 3) number of layers with different acoustic properties the beam travels across. The quality of an US scan is enhanced as the target is closer to the US probe. In the particular case of lung scanning, directing beams through air should be avoided due to the scattering phenomenon, which significantly reduces the interpretability of the resulting scan. Skeletal structures reflect US almost entirely, prompting the user to avoid them at all cost. Last, layers of medium with different attenuation coefficients induce additional refraction and reflection of the signal, negatively impacting the imaging outcomes.

The problem was therefore formulated as a discrete optimization solved by linear search, whereby the objective is to minimize the sum of weights assigned to various structures in the human body through which the US beam travels, along with interaction terms modeling refraction, reflection, and attenuation of the signal. Let ${\vec{p}}_{i}\in {\mathbb{R}}^{2}$ represent the 2D coordinates of a pixel *i* inside the ultrasound beam cone (see Figure 3 for cone reference). Let ${\vec{p}}_{fc}\in {\mathbb{R}}^{2}$ represent the pixel corresponding to the focal point of the ultrasound probe. The attenuation of the ultrasound signal is evaluated through the following equation (Narayana et al., 1984):

$$w_{i,c}=w_{0,c}{\left[\text{exp}\left(-\alpha \text{Δ}||{\vec{p}}_{i}-{\vec{p}}_{fc}|{|}_{2}\right)\right]}^{-1},$$ (1)

whereby $w_{0,c}$ is the weight of the first pixel pertaining to the same class *c*, α is the attenuation coefficient of the medium, and Δ is the spatial resolution of the CT scan. To model the intensity reflection of the ultrasound beam at the interface of two different mediums, first the intensity reflection coefficient γ is evaluated, and subsequently applied to the weight of the first pixel following the interface boundary (Laugier and Haïat, 2011):

$$\gamma =\frac{{\left(\rho_{2}v_{2}-\rho_{1}v_{1}\right)}^{2}}{{\left(\rho_{2}v_{2}+\rho_{1}v_{1}\right)}^{2}},$$ (2)

$$w_{i,c}^{\text{*}}=w_{i,c}{\left(\gamma \right)}^{-1},$$ (3)

with ρ being tissue density and *v* the speed of ultrasound. The term ρv effectively represents the impedance of the medium, with medium 1 preceding medium 2. The algorithm thus evaluates the weight of every pixel in between the first point of contact of the ultrasound probe and the target point, with higher weights assigned to more attenuated pixels, as they would drastically reduce the image quality. The US path that results in the lowest weight is selected as the optimal one.

To this end, bones were assigned the highest weight of $10^{9}$, since US rays cannot travel past them. The second highest weight was assigned to lung air at 5, followed by soft tissues at 1. These weights were derived with the assistance of an expert sonographer. The assumed attenuation coefficients for skeletal tissue, lung air, and soft tissues are 1.1 dB/(mm × MHz), 1.2 dB/(mm × MHz), and 0.12 dB/(mm × MHz), respectively. The assumed densities of each class are 2000 kg/m^3^, 1.225 kg/m^3^, and 1,000 kg/m^3^, whereas the speed of sound is 3,720 m/s, 330 m/s, and 1,575 m/s, respectively (Zagzebski, 1996; Etchison, 2011; Shung, 2015; Szalma et al., 2019). The weights are computed within an US cone that can only be instantiated from the surface of the patient’s body across all generated images. The algorithm first determines the optimal scanning position and orientation of the probe for each individual image, and then selects the image with the overall lowest returned weight. The position of the US cone, as well as the orientation of the image, defines the optimal US scanning position and orientation in the CT coordinate frame. The solution was deployed on the Alienware laptop used for the robot control, and a pseudo-code is provided for reference in **Algorithm 1**.

Algorithm 1: Ultrasound Probe Optimal Scanning Position and Orientation

1: input: CT scan, target point ${\vec{p}}_{t}\in {\mathbb{R}}^{3}$, angle resolution Δβ=5, Δ in mm
2: procedure
3: extract N×M image $I_{0}$ from CT in axial plane s.t. ${\vec{p}}_{t}\in I_{0}$
4: extract $N^{\prime }\times M^{\prime }$ images $I_{i}$ from CT in tilted planes at angles Δβ s.t. ${\vec{p}}_{t}\in I_{i}$
5: generate background masks $M_{BK,i}$ for $I_{i},i:0\to k$
6: generate lung air masks $M_{L,i}$ for $I_{i},i:0\to k$
7: generate bones masks $M_{BN,i}$ for $I_{i},i:0\to k$
8: generate soft tissue masks $M_{T,i}$ for $I_{i},i:0\to k$
9: replace $I_{i}$ with. $M_{i}:=M_{BK,1}+M_{T,i}+M_{L,i}+M_{BN,i},i:0\to k$
10: initialize empty weight vector $W\in {\mathbb{R}}^{\left(k+1\right)\times L}$
11: For i:=0 to *k* do
12: generate *L* US beam cone contours *C* for $I_{i}$ *s.t.* ${\vec{p}}_{t}\in A_{l}$, $A_{l}$: = area enclosed by contour
13: **For** j:=0 **to** L−1 **do**
14: define. $\vec{d}:={\vec{p}}_{t}-{\vec{p}}_{fc}$
15: define ${\vec{p}}_{closest}:=\text{argmin}\left(||{\vec{p}}_{US}-{\vec{p}}_{fc}|{|}_{2}\right)$ *s.t.* ${\vec{p}}_{US}\in \vec{d}$
16: reinitialize $\vec{d}:={\vec{p}}_{closest}-{\vec{p}}_{t}$
17: define vector $\vec{p}:={\left[M_{i}\left[{\vec{p}}_{closest}\right],\ldots ,M_{i}\left[{\vec{p}}_{t}\right]\right]}^{T}$ that contains all pixel values along $\vec{d}$
18: initialize $w=\vec{p}\left[0\right]$
19: initialize $w_{0,T}=1$, $w_{0,L}=5$ and $w_{0,B}=10^{9}$
20: For t:=1 to length ($\vec{p}$) do
21: If $\vec{p}\left[t-1\right]\neq \vec{p}\left[t\right]$do
22: $\vec{p}\left[t:end\right]=\vec{p}\left[t:end\right]{\left[\frac{{\left(\rho_{t}v_{t}-\rho_{t-1}v_{t-1}\right)}^{2}}{{\left(\rho_{t}v_{t}+\rho_{t-1}v_{t-1}\right)}^{2}}\right]}^{-1}$
23: update $w_{0,c}$ according to $\vec{p}\left[t:end\right]$ for corresponding classes
24: $w=w+w_{0}{\left[\text{exp}\left(-\alpha_{C}\text{Δ}||{\vec{p}}_{i}-{\vec{p}}_{closest}|{|}_{2}\right)\right]}^{-1}$
25: append *w* to *W*
26: return by linear search *w_min_* *:* = *Argmin* W
27: return ${\vec{p}}_{goal}\in {\mathbb{R}}^{3}$ corresponding to $w_{min}$ evaluated from plane transformation
28: return $R_{goal}\in SO\left(3\right)$ corresponding to $w_{min}$ evaluated from plane transformation
29: output: *R_goal_* *and* w_min_

### 2.4 Force–Displacement Profiles Along the Ribcage

Uncertainties in patient registration and robot kinematics can result in a partial or complete occlusion of the region of interest due to the misplacement of the US probe. To mitigate this problem, a force–feedback mechanism is proposed, whereby we hypothesize that for a constant force application of 20 N, which is the recommended value for abdominal US imaging (Smith-Guerin et al., 2003), the probe’s displacement within the phantom body will be higher in-between the ribs as opposed to being on the ribs. If the hypothesis is validated, one can thus generate a displacement profile across the ribcage of a patient to detect the regions obstructed by the ribs. The displacement on the phantom is expected to follow a sinusoidal profile, with peaks (i.e., largest displacements) corresponding to a region in-between the ribs, and troughs (i.e., smallest displacement) corresponding to a region on the ribs.

To evaluate this hypothesis, we have generated solid models of *n* = 3 patient torsos using anonymized CT scans, which were used to simulate displacements using finite element analysis (FEA) in ANSYS (ANSYS, Canonsburg, Philadelphia, United States). All image data are stripped from patient-identifying information, and are thus concordant with the exempt status described in 45 CFR §46.102. Two of the patients are female. The third patient is a male and was used as the model for the phantom’s creation. All patients have varying BMI. The different organs of the patients were extracted from the CT scans using Materialize Mimics (Materialize NV, Southport, QLD 4222, Australia) software as STL files, and subsequently converted through SOLIDWORKS (SolidWorks Corp., Dassault Systemes, Velizy-Villacoublay, France) into IGS format, thus transforming the mesh surfaces into solid objects that can undergo material assignment and FEA simulations. The tissues’ mechanical properties for the female patients were obtained from the literature, whereas those of the phantom were measured experimentally (Al-Zogbi et al., 2020). In the FEA simulations, the force was transmitted onto the bodies through a CAD model of the ultrasound probe. In the robotic implementation, the ultrasound probe does not slip on a patient’s body when contact is established because of the setup’s rigidity. A lateral displacement of the probe in simulation would be erroneously translated into soft tissue displacement since the probe’s tip displacement was used to represent the soft tissue displacement. Thus to ensure that the motion of the probe is confined to a fixed vector, the probe’s motion was locked in all directions except in the *z*-axis. The force was directly applied to the probe through a force load that gradually increases from 0 to 20 N over a period of 5 s. The probe was initially positioned at a very close proximity from the torso; hence, its total displacement was considered to be a measure of the tissue’s displacement. The simulations were deployed on a Dell Precision workstation 3620 with an i7 processor and 16 GB of RAM. Each displacement data point required on average 2.5 h to converge.

**FIGURE 4:**
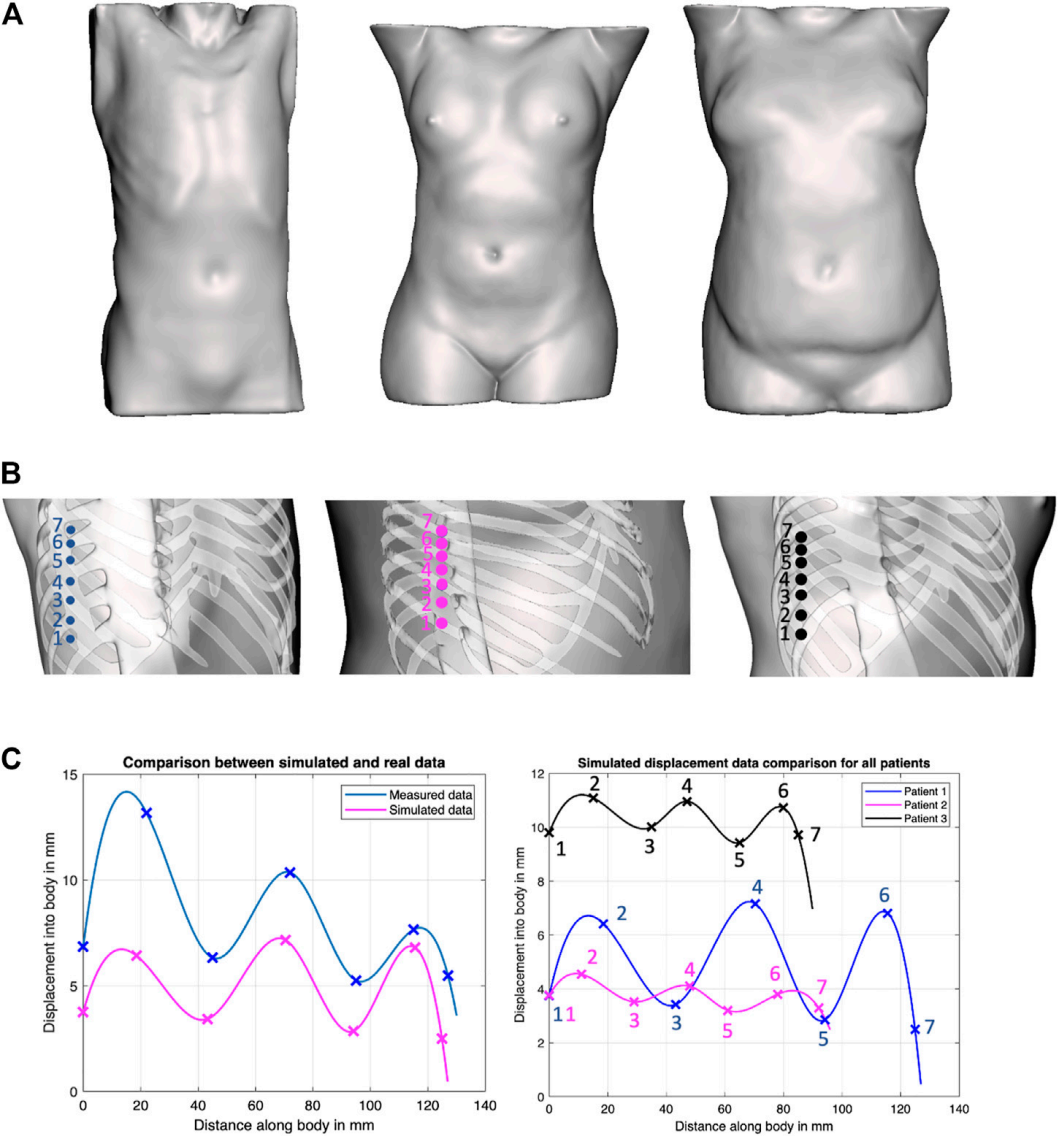
Force–displacement validation experiments. **(A)** Surface models of the three patients considered in our work, covering both genders and various BMI; **(B)** positions on the phantoms where displacement profiles were collected in ANSYS simulations; **(C)** Comparison of experimental results between simulated data and actual phantom (patient 1, left plot), and comparison of simulated data between all patients (right plot).

The location of the collected data points, as well as the returned displacement profile for all the three patients, is shown in Figure 4. Cubic splines were used to fit the data to better visualize the trend using MATLAB’s curve fitting toolbox. Cubic splines were considered for better visualizing the profiles, assuming a continuous and differentiable function to connect data points. The actual displacements in between the minimum and maximum, however, need to be further validated, and may not match the displayed spline. To verify the outcome of the simulations, the corresponding displacement profiles were collected from the physical phantom, which are reported in Figure 4 as well. Although the physical test demonstrates overall larger displacements, the physical displacement trend is similar to those of the simulated experiments. The results thus provide preliminary validation of our hypothesis of varying displacements associated with different positioning of an US probe with respect to the ribs. Therefore, we adopted this strategy in the robot’s control process, whereby the system collects several displacement data points around the goal position at 20 N, to ensure that the US image is obtained in-between the ribs.

### 2.5 Ribcage Landmark Prediction

This framework uses 3D landmarks defined on the ribcage to estimate the optimal probe’s position. We trained a deep convolutional neural network to estimate the 3D position of 60 landmarks on the ribcage from the skin surface data. We define these landmarks using the segmentation masks of ribs obtained from the CT data. From the segmentation masks, we compute the 3D medial axis for each rib using a skeletonization algorithm (Lee et al., 1994). The extremities and center of each rib for the first 10 rib pairs (T1 to T10) are used as landmark locations. The three landmarks thus represent the rib–spine intersection, the center of the rib, and the rib–cartilage intersection.

Recent research in deep learning has shown encouraging progress in detecting 3D landmarks from surface data (Papazov et al., 2015; He et al., 2019; Liu et al., 2019). Teixeira et al. (2018) introduced a method to estimate 2D positions of internal body landmarks from a 2.5D surface image of the torso generated by orthographically projecting the 3D skin surface. Such methods, however, do not generalize to 3D data, since it would require representing landmark likelihood as a Gaussian distribution over a dense 3D lattice, and the memory required for representing 60 such lattices, one for each landmark, would be overwhelming. We address this by training a 3D deep convolutional network to directly estimate the landmark coordinates in 3D from the 3D volumetric mask representing the skin surface of a patient’s torso. Given the skin mask, we estimate a 3D bounding box covering the thorax region, using jugular notch on the top and pelvis on the bottom. We then crop this region and resize to 128 × 128 × 128 volume, which is used as input to a deep network. The network outputs a 3 × 60 matrix, which represents the 3D coordinates of the 60 rib landmarks. We adopt the DenseNet architecture (Huang et al., 2016) with batch normalization (Ioffe and Szegedy, 2015), and LeakyReLU activations (Xu et al., 2015) with a slope of 0.01 following the 3D convolutional layers with kernel size of 5x5x5. The network parameters were optimized using AdaDelta (Zeiler, 2012).

### 2.6 Control Strategy for Skeletal Structure Avoidance

**FIGURE 5:**
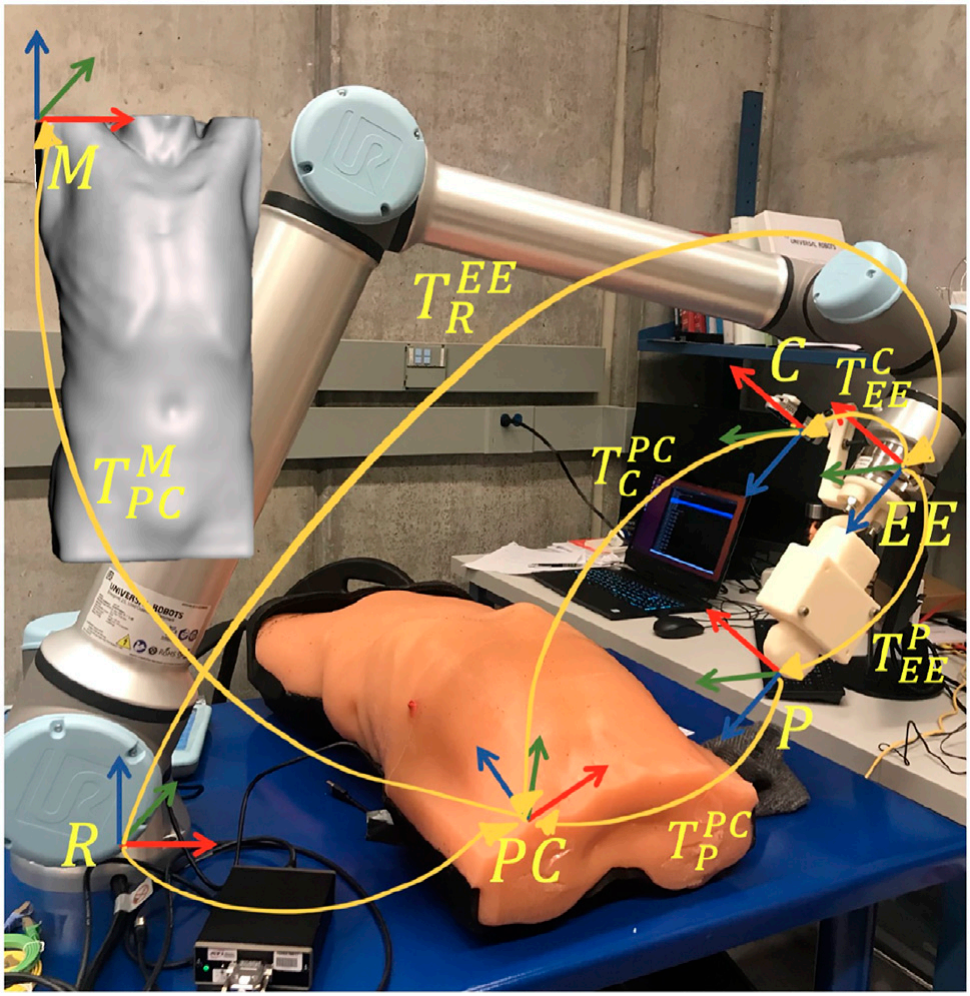
Overview of the different reference frames used in the implementation, as well as the homogeneous transformations between them.

The autonomous positioning of the US probe in contact with the patient’s body necessitates motion and control planning. Let $T_{B}^{A}$ denote the homogeneous transformation matrix from frames *A* to *B*, composed of a rotation matrix $R_{B}^{A}\in SO\left(3\right)$, and a translation vector ${\vec{p}}_{B}^{A}\in {\mathbb{R}}^{3}$. The global reference frame for the robotic implementation was chosen as the base frame of the robot, denoted by frame *R*. Let *C* and *p* represent the frames attached to the camera and tip of the US probe, as shown in Figure 5. Since both camera and probe are rigidly affixed to the robot’s end effector, $T_{R}^{C}$ and $T_{R}^{P}$ are constant. $T_{R}^{C}$ is estimated by performing an *eye-in-hand* calibration following the scheme presented in Tsai and Lenz (1989), whereas $T_{R}^{P}$ is evaluated from the CAD model of the probe and its holder. Note that these transformations are composed of two transformations, namely,

$$T_{R}^{C}=T_{R}^{EE}T_{EE}^{C},$$ (4)

$$T_{R}^{P}=T_{R}^{EE}T_{EE}^{P},$$ (5)

whereby EE corresponds to the robot’s end effector frame. The holder is designed such that the frame of the US probe would be translated by a fixed distance along the *z*-direction of the manipulator’s end effector frame (see Figure 5). Thus, in the physical workspace, the relationship used to map out the point cloud data (frame PC) to the robot’s base frame is

$$T_{R}^{PC}=T_{R}^{C}T_{C}^{PC}.$$ (6)

The anonymous CT patient scans (frame CT) were used to generate the mesh model (frame *M*) of the torsos, and hence the transformation between the two is known, set as $R_{CT}^{M}=I$ and ${\vec{p}}_{CT}^{M}={\left[0,0,0\right]}^{T}$. The transformation $T_{PC}^{M}$ between the point cloud data and the mesh model is estimated through the pointmatcher library using the iterative closest point approach (Pomerleau et al., 2013). Initial target points are either defined in the CT scans, or on the mesh models, both of which correspond to the *M* frame. Since the US probe target position and orientation need to be defined in the US probe frame (*p*), the following transformation is used:

$$T_{P}^{M}=T_{R}^{P-1}T_{R}^{C}T_{C}^{PC}T_{PC}^{M}.$$ (7)

The overall control algorithm can be described through three major motion strategies: 1) positioning of the US probe near the target point, 2) tapping motion along the ribcage in the vicinity of the target point to collect displacement data at 20 N force, and 3) definition of an updated scanning position to avoid ribs, followed by a predefined sweeping motion along the probe’s *z*-axis.

The trajectory generation of the manipulator is performed by solving for the joint angles $\theta_{i},i\in \left[0,5\right]$ through inverse kinematics computation facilitated by Open Robotics Control Software (OROCOS), and the built-in arm controller in the robot driver. Since obstacle avoidance has not been explicitly integrated into the robot’s motion generator, we have defined a manipulator home configuration, from which the system can reach various target locations without colliding with the patient and the table. The home configuration is centered at the patient’s torso at an elevation of 0.35°m from the body, with the +z axis of the end effector corresponding to the −z axis of the robot’s base frame. The robot is driven to the home configuration before each target scan.

The force–displacement collection task begins with the robot maintaining the probe’s orientation fixed (as defined by the goal), and moving parallel to the torso at regular intervals of 3 mm, starting at 15 mm away from the goal point, and ending 15 mm past the goal point, resulting in a total of 11 readings. The robot thus moves along the end effector’s +z-axis, registering the probe’s position when a force reading is first recorded, and when a 20 N force is reached. The $L_{2}$ norm of the difference of these positions is stored as a displacement data point. The two data points that represent the smallest displacements are assumed to be rib landmarks, and represent the center of the corresponding rib. The ideal direction of the applied force would be normal to the centerline of the curved section of the probe; however, it may not always be the case as some regions in the lungs might only be reachable with the resultant force pushing the probe on the side. In the case where the measured lateral forces contributed to the overall force by over 20%, the overall force was then considered in the computations. The center of mass of the probe holder is not in line with the assumed center; however, it is stiff enough to prevent bending.

Since the goal point is always located between two ribs, it can hence be localized with respect to the center of the two adjacent ribs. The goal point is thus projected onto the shortest straight segment separating the center of the ribs, which is also closest to the goal point itself. Let the distance of the goal point from Rib 1 be $d_{1}$. Since the ribs are fairly close to each other, a straight line connecting the two is assumed to avoid modeling the curvature of a torso. Once two points with the smallest displacement are identified from the force collection procedure, a line connecting the two is defined in the end effector’s coordinate frame, and distance $d_{1}$ is computed along that line from Rib 1 to define the position of the updated target point. Maintaining the same optimal orientation, the robot is thus driven to the updated goal point, the end effector then moves along the probe’s +z-axis until a 20 N force is reached, followed by a sweeping motion of $\pm 30^{\circ }$ around the probe’s line of contact with the patient.

## 3 Experiments and Results

### 3.1 Scanning Points Detection

**FIGURE 6:**
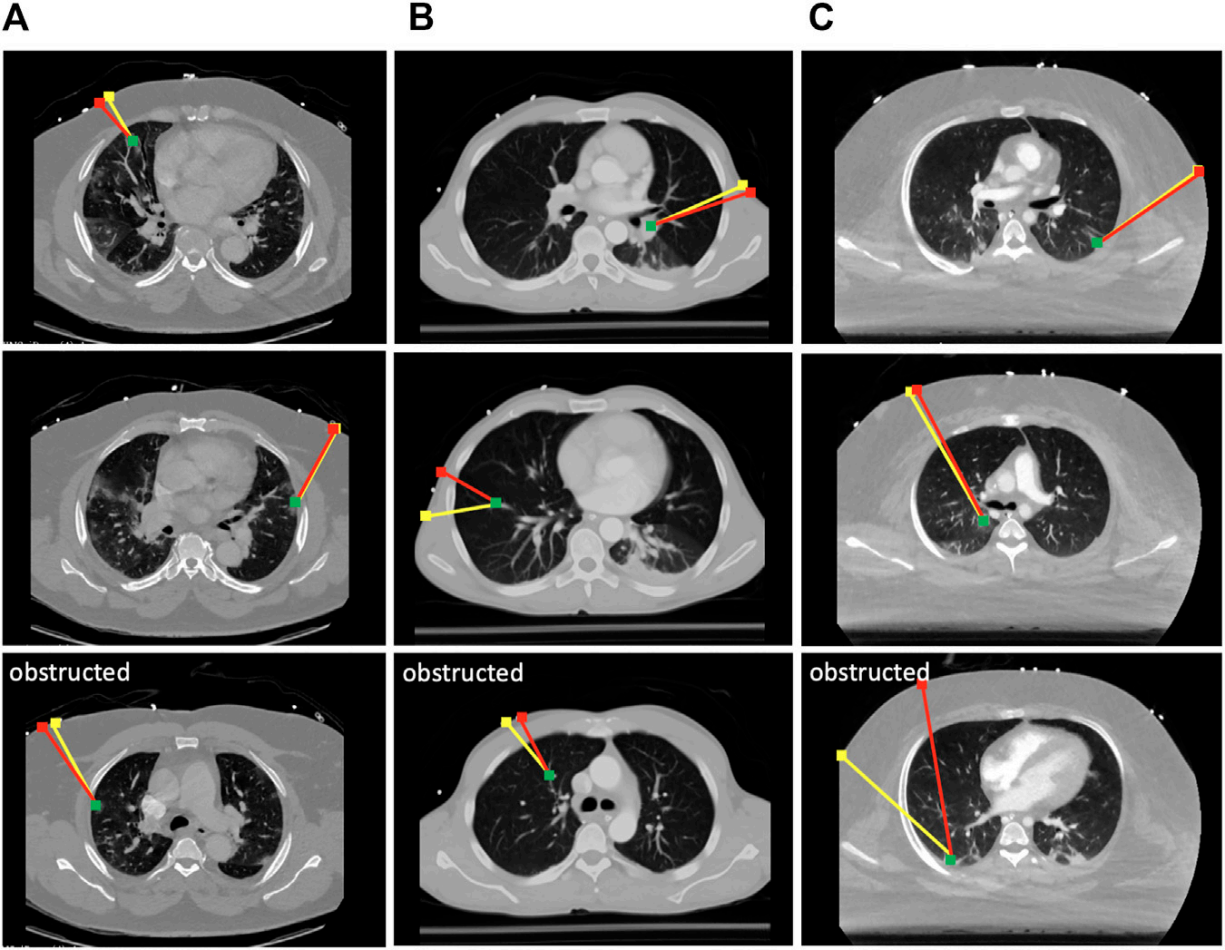
Selected results from the optimal scanning algorithm compared to medical expert’s selection. The yellow lines mark the expert’s proposed paths, and the red lines are the ones proposed by the algorithm. The green dots are the target points that need to be imaged in the lungs. Paths returned by the expert that were obstructed by the ribs are marked as such. Each column of results corresponds to a CT scan of a different patient. **(A)** Results from COVID-19 patient 1; **(B)** results from a healthy patient; **(C)** results from COVID-19 patient 2.

To evaluate the effectiveness of the scanning point detection algorithm, we asked an expert radiologist to propose scanning points on the surface of *n* = 3 patients using CT data in 3D Slicer. All image data are stripped from patient-identifying information, and are thus concordant with the exempt status described in 45 CFR §46.102. Two of the patients were positive for COVID-19 and exhibited significant infiltrate formation in their lungs, whereas the last patient was healthy with no lung abnormalities (see Figure 6). The medical expert selected 10 different targets within the lungs of each patient at various locations (amounting to a total of 30 data points), and proposed corresponding probe position and orientation on the CT scans that would allow them to image the selected targets through US. The medical expert only reviewed the CT slices along the main planes (sagittal, transverse, and coronal). The following metrics were used to compare the expert’s selection to the algorithm’s output: 1) bone obstruction, which is a qualitative metric that indicates whether the path of the US center beam to the goal point is obstructed by any skeletal structure; and 2) the quality of the US image, which was estimated using the overall weight structure discussed in the previous section, whereby a smaller scan weight signals a better scan, with less air travels, scattering, reflection, and refraction. For a fair initial comparison, we restricted the image search in the detection algorithm to the plane considered by the medical expert, i.e., the optimal scanning point was evaluated across a single image that passes through the target point. In this setting, the algorithm did not return solutions that were obstructed by bones, whereas five out of 30 of the medical expert’s suggested scanning locations resulted in obstructed paths, as shown in Figure 6.

The quality of the scans has been compared on the remaining unobstructed 25 data points, and it was found that the algorithm returned paths with an overall 6.2% improvement in US image quality as compared with the expert’s selection based on the returned sum of weights. However, when the algorithm is reset to search for optimal scanning locations across several tilted 2D images, the returned paths demonstrated a 14.3% improvement across the 25 data points, indicating that it can provide estimates superior to an expert’s suggestion that was based on exclusively visual cues. The remaining five points have also been tested on the algorithm, and the optimal scanning locations were successfully returned. The average runtime for the detection of a single optimal scanning position and orientation is 10.5±2.10 min, evaluated from the aforementioned 30 target points. The two most time-consuming tasks are the generation of oblique planes from the CT scans, and the Gustafson–Kessel clustering used to delineate lung air. Since this is a preprocessing step, the rather large time consumption is not a concern.

### 3.2 Ribcage Landmark Prediction

**FIGURE 7:**
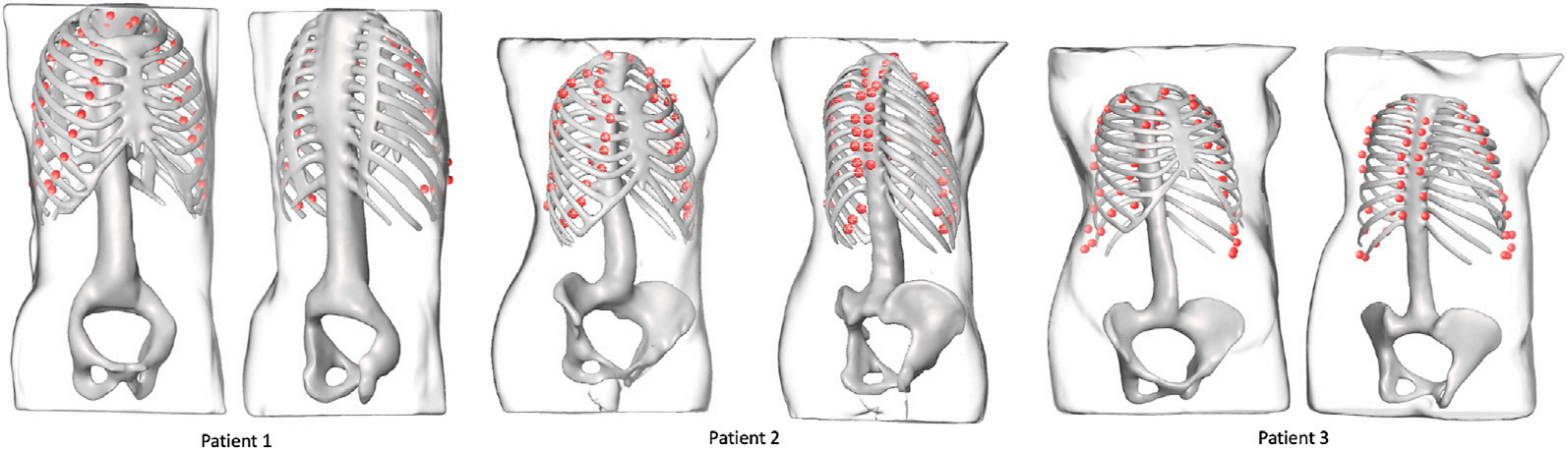
Landmark outputs for every patient superimposed on the ribcages. The ribcage acts as ground truth for bone detection using the proposed landmarks. The skeletons are used for visualization purposes and were not part of the training process.

**FIGURE 8:**
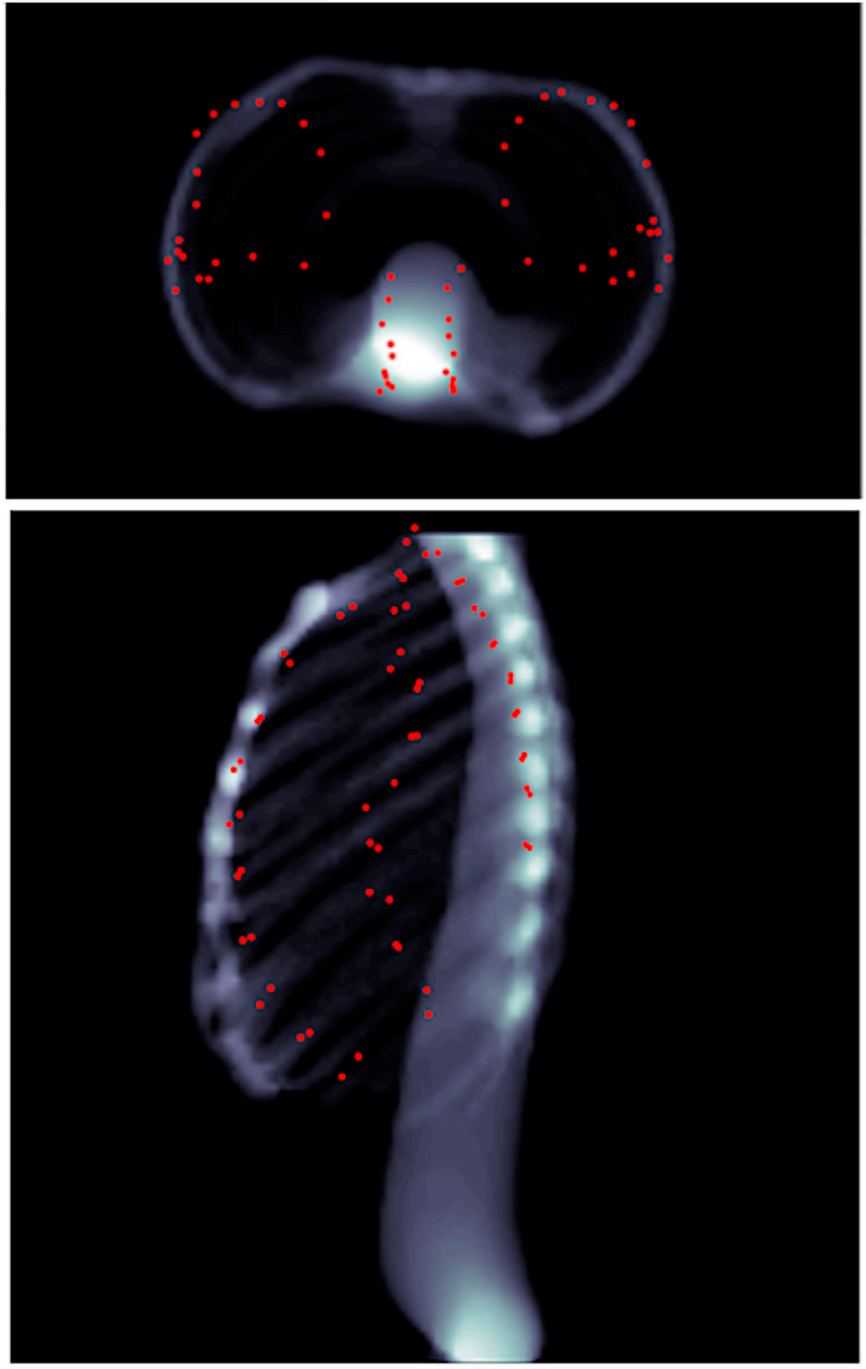
Predicted landmarks (represented by red points) on patient 1 as seen through head–feet projection **(top)**, as well as lateral projection **(bottom)**. The landmarks are not part of the lungs, but merely appear so because of the projection.

A total of 570 volumes were prepared from thorax CT scans, 550 of which were used for training and 20 for testing. All image data are stripped from patient-identifying information and are thus concordant with the exempt status described in 45 CFR §46.102. Each of the 570 volumes is obtained from a different patient. In the training set, the minimum ribcage height was 195 mm and the maximum height was 477 mm, whereas in the testing set the minimum ribcage height was 276 mm and the maximum height was 518 mm. The percentile distribution of the training and testing ribcage heights is detailed in Table 1. The network was trained for 150 epochs optimizing the $L_{1}$ distance between the predicted coordinates and the Ground truth coordinates using the Adam optimizer (Kingma and Ba, 2015). The training took place on an NVIDIA Titan Xp GPU using the PyTorch framework (Paszke et al., 2017) and converged in 75 min. A mean Euclidean error of 14.8 ± 7.00 mm was observed on the unseen testing set, with a 95th percentile of 28 mm. The overall inference time was on average 0.15 s. Figure 7 shows the landmark predictions obtained using the trained model on the three human subjects discussed in Section 2.4, by taking their corresponding skin surface masks as input. Figure 8 shows the projected landmarks in 2D images.

**TABLE 1:** Percentile distribution of the ribcage heights for training and testing the 3D landmark prediction algorithm.

	Percentile								
Ribcage height (mm)	10^th^	20^th^	30^th^	40^th^	50^th^	60^th^	70^th^	80^th^	90^th^
Training set	282	293	304	321	344	372	388	407	428
Testing set	337	351	363	371	379	399	407	432	447

### 3.3 Evaluation of Robotic System

A total of four experiment sets were devised to analyze the validity of our two main hypotheses, which involve the evaluation of the robotic system: 1) with prior CT scans without force feedback, 2) with prior CT scans with force feedback, 3) with ribcage landmark estimation without force feedback, and 4) with ribcage landmark estimation with force feedback. The overall performance of the robotic system is assessed in comparison with clinical requirements, which encompass three major elements: 1) prevention of acoustic shadowing effects whereby the infiltrates are blocked by the ribcage (Baad et al., 2017); 2) minimization of distance traveled by the US beam to reach targets, particularly through air (Baad et al., 2017); and last 3) maintaining a contact force below 20 N between the patient’s body and US probe (Smith-Guerin et al., 2003).

#### 3.3.1 Simulation Evaluation

Due to the technical limitations imposed by the spread of COVID-19 itself, the real-life implementation of the robotic setup was limited to *n* = 1 phantom. Additional results are thus reported using Gazebo simulations. The same three patients described in Section 2.4 are used for the simulation. Since Gazebo is not integrated with an advanced physics engine for modeling tissue deformation on a human torso, we replaced the force sensing mechanism with a ROS node that compensates for the process of applying a force of 20 N and measuring the displacement of the probe through a tabular data lookup, obtained from the FEA simulations. In other words, when the US probe in the simulation approaches the torso, instead of pushing through and measuring the displacement for a 20 N force (which is not implementable in Gazebo for such complex models), we fix the end effector in place, and return a displacement value that was obtained from prior FEA simulations on the corresponding torso model.

**FIGURE 9:**
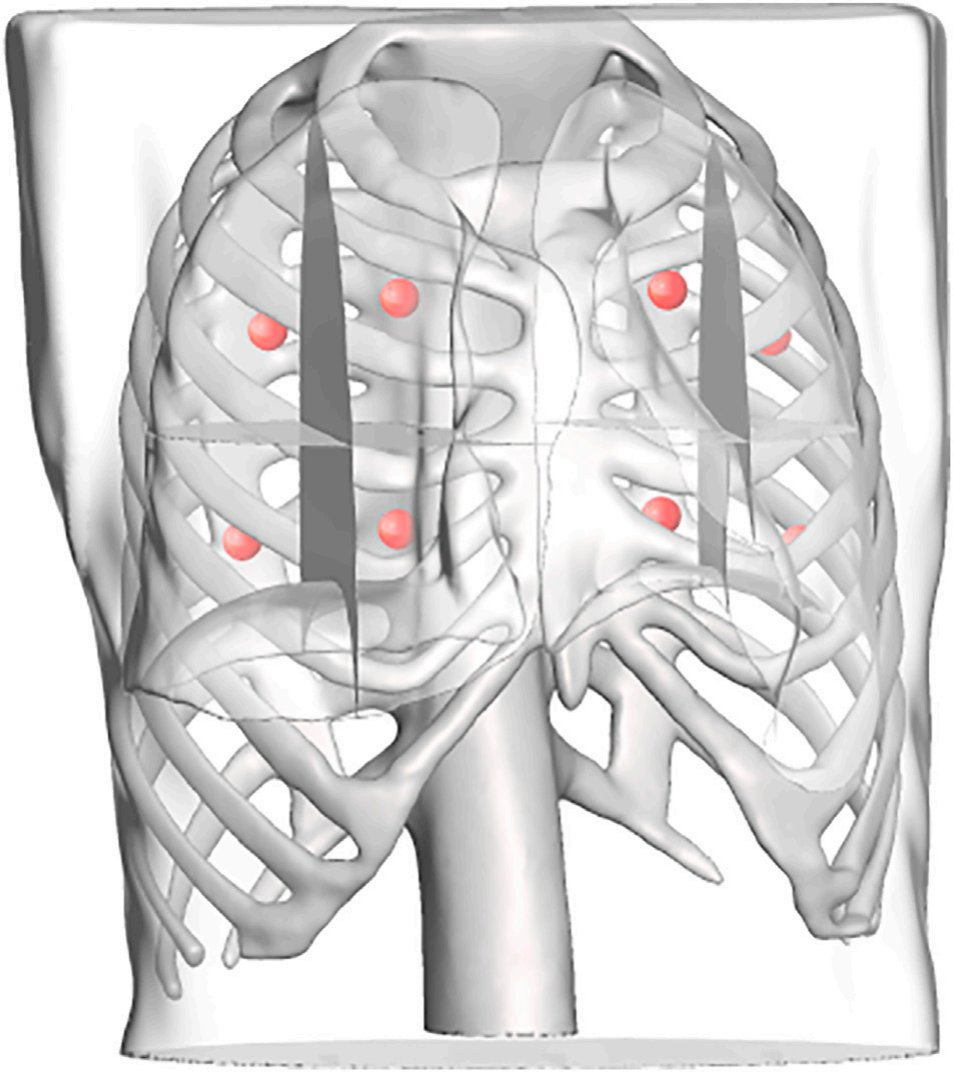
Each lung is divided into four regions, the centroids of which are computed and used as target points in the scanning point detection algorithm.

To replicate a realistic situation with uncertainties and inaccuracies, the torso models are placed in the simulated world at a predefined location, corrupted with noise in the *x*, *y*, and *z* directions, as well as roll, pitch, and yaw angles. Errors were estimated based on reported camera accuracy, robot’s rated precision, and variations between original torsos design and final model. The noises were sampled from Gaussian distributions with the precomputed means, using a standard deviation of 10% of the mean. The numerical estimates on the errors are reported in Table 2. The exact location of the torsos is thus unknown to the robot. For each torso model, a total of eight points were defined for the robot to image, four on each side. Each lung was divided into four quadrants, and the eight target points correspond to the centroid of each quadrant (see Figure 9). This approach is based on the 8-point POCUS of lungs. The exact location of the torso is used to assess the probe’s position with respect to the torso, and provide predicted US scans using CT data. Two main evaluation metrics are considered: 1) the positional accuracy of the final ultrasound probe placement, which is the $L_{2}$ norm of the difference between the target ultrasound position, and the actual final ultrasound position; and 2) a binary metric for imaging the goal infiltrate or region, whereby it is either visualized or obstructed.

**TABLE 2:** Elements contributing to the overall error estimation. RPY stands for roll, pitch, and yaw. All values in the x,y,z directions are reported in mm, and the RPY values are in radians.

	x	y	z	R	*p*	Y
Registration	5	5	30	0.10	0.10	0.10
Robot	0.1	0.1	0.1	0.01	0.01	0.01
Model	10	10	10	0.05	0.05	0.05
Total	∼15	∼15	∼40	0.16	0.16	0.16

Each experiment set was repeated 10 times with different sampled errors at every iteration. The results of all four experiment sets are reported in Table 3. It is noticeable that the force feedback component has decreased the overall error on the probe’s placement by 49.3% using prior CT scans, and 52.2% using predicted ribcage landmarks. The major error decrease was observed along the *z*-axis, since the force feedback ensured that the probe is in contact with the patient. It also provided additional information on the rib’s placement near the target points, which were used to define the same target points’ positions relative to the rib’s location as well. For the US probe placement using only force–displacement feedback, we can observe that the average error across all three models is 20.6 ± 14.7 mm. Using the final probe’s placement and orientation on the torso, we converted these data into CT coordinates to verify that the point of interest initially specified was imaged. In all of the cases, the sweeping motion allowed the robot to successfully visualize all the points of interest. When using ribcage landmark estimation, the displacement error with force feedback for all the three patients averaged at 19.8 ± 16.9 mm. Similarly, the data were transformed into CT coordinates, showing that all the target points were also successfully swept by the probe. The average time required for completing an 8-point POCUS scan on a single patient was found to be 3.30 ± 0.30 min and 18.6 ± 11.2 min using prior CT scans with and without force feedback, respectively. The average time for completing the same scans was found to be 3.80 ± 0.20 min and 20.3 ± 13.5 using the predicted ribcage landmarks with and without force feedback, respectively. The reported durations do not include the timing to perform camera registration to the robot base, as it is assumed to be known a priori.

**TABLE 3:** Probe positioning error evaluation on the simulation experiments for the three patients. Experiments are divided by cases; case 1: using CT data without force feedback; case 2: using CT data with force feedback; case 3: using landmark prediction only without force feedback; case 4: using landmark prediction only with force feedback. All values are reported in mm.

	Case 1	Case 2	Case 3	Case 4
Patient 1				
Average error in x	13.7	9.40	16.5	12.3
Average error in y	12.7	10.1	14.6	6.70
Average error in z	30.3	11.5	31.2	8.10
Total error	35.5	17.9	38.1	16.1
Standard deviation	18.2	12.8	18.5	16.8
Patient 2				
Average error in x	14.2	13.2	12.4	11.2
Average error in y	10.5	8.70	15.3	13.4
Average error in z	41.0	13.5	35.6	15.1
Total error	44.6	20.7	40.6	23.0
Standard deviation	23.4	14.5	9.80	16.2
Patient 3				
Average error in x	17.8	15.0	19.8	11.3
Average error in y	13.6	13.9	12.4	10.9
Average error in z	35.6	11.1	39.1	13.2
Total error	42.0	23.2	45.5	20.5
Standard deviation	18.7	16.7	21.4	17.6

#### 3.3.2 Phantom Evaluation

**FIGURE 10:**
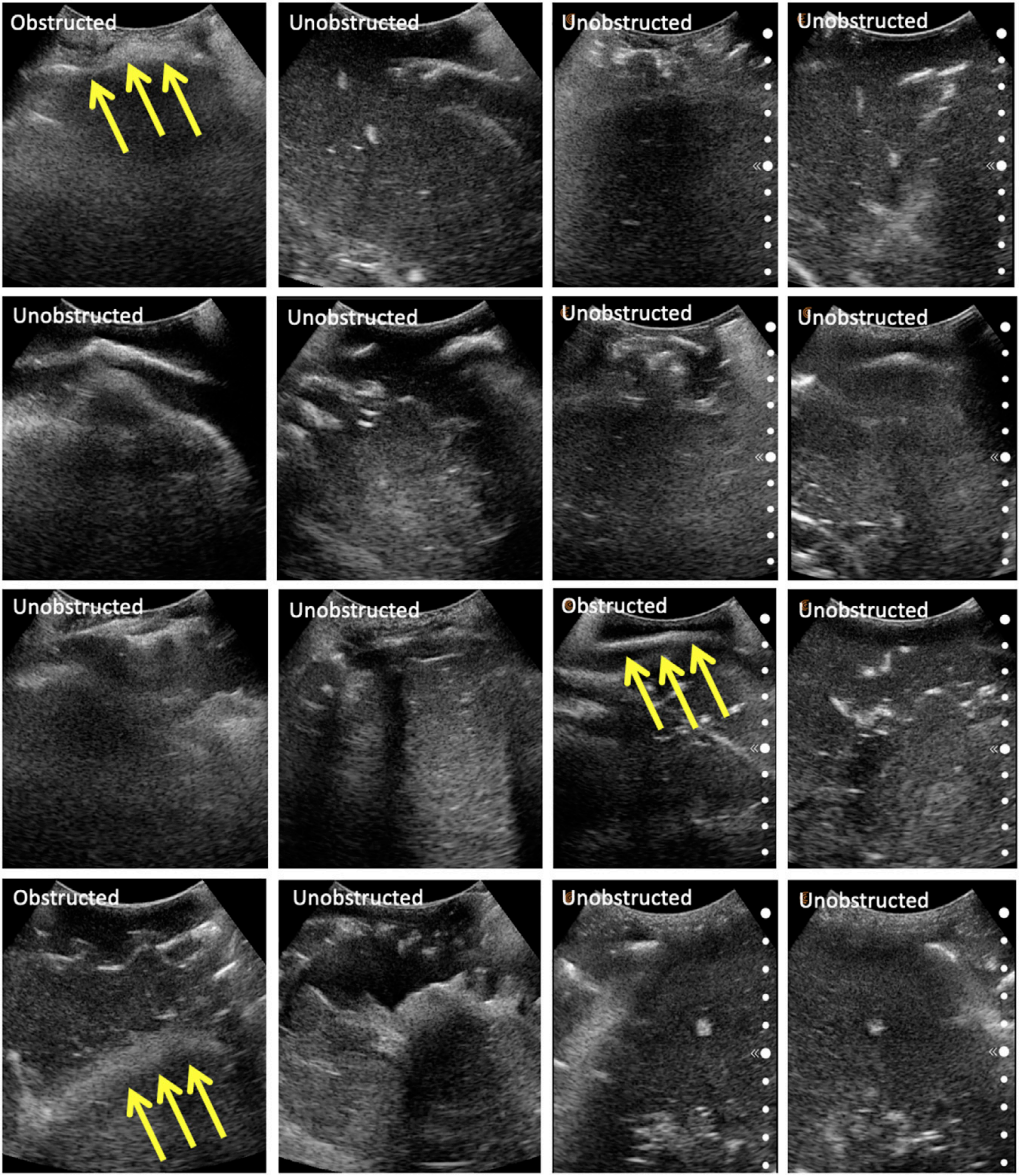
Selected US scans obtained from the phantom through the different sets of experiments. Scans that are obstructed by bones are marked as such, with the arrows pointing toward the bone. Each column represents a different experiment, namely: **(A)** scans using CT data without force feedback; **(B)** scans using CT data with force feedback; **(C)** scans using landmark prediction only without force feedback; **(D)** scans using landmark prediction only with force feedback.

The same eight points derived from the centroids of the lung quadrants were used as target points for the physical phantom. Since the manufactured phantom does not contain lungs or visual landmarks, we are evaluating the methodology qualitatively, categorizing the images into three major groups: 1) completely obstructed by bones whereby 50–100% of the field of view is uninterpretable, with the goal point shadowed; 2) partially obstructed by bones, whereby <50% of the field of view is uninterpretable, with the goal point not shadowed; and 3) unobstructed, whereby <10% of the field of view is interpretable, and the goal point not shadowed. These groupings were developed in consultation with an expert radiologist. Since the scanning point algorithm focuses on imaging a target point in the center of the US window, we are also reporting this metric for completeness. The phantom was assessed by an expert radiologist, confirming that the polycarbonate from which the ribcage is made is clearly discernible from the rest of the gelatin tissues. It does, however, allow for the US beam to traverse it, meaning that the “shadow” resulting from the phantom’s rib obstruction will not be as opaque as that generated by human bones. The robot manipulator is first driven to the specified goal point, and displacement profiles are collected in the vicinity of the target. The ribs’ location is estimated from the force–displacement profile, and the final goal point is recomputed as a distance percentage offset from one of the ribs. Each experiment set was repeated three times, the results of which are reported in Table 4. The evaluation of the US images was performed by an expert radiologist. The results show that the force feedback indeed assists with the avoidance of bone structures for imaging purposes, whereby 100% of the US scans using prior CT data were interpretable (i.e., with a visible center), and 87.5% of the scans were interpretable using landmark estimation. The results without using force feedback show that only 75% of the scans have a visible center region using prior CT scans, and 58.3% using predicted landmarks. The landmark estimation approach demonstrated worse results due to errors associated with the prediction, which can cause erroneous ribs’ location estimation, and thus bad initial target points suggestions. Select images for all four experiments are shown in Figure 10. The average time required for completing an 8-point POCUS scan on the phantom was found to be 3.40 ± 0.00 min and 17.3 ± 5.10 min using prior CT scans with and without force feedback, respectively. The average time for completing the scans was found to be 3.30 ± 0.00 min and 25.8 ± 7.40 min using predicted ribcage landmarks with and without force feedback, respectively. Camera registration time was not included in reported durations.

**TABLE 4:** Qualitative categorization of all US images as evaluated by an expert radiologist. The US images were collected from the phantom. Experiments are divided by cases; case 1: using CT data without force feedback; case 2: using CT data with force feedback; case 3: using landmark prediction only without force feedback; case 4: using landmark prediction only with force feedback.

	Completely obstructed	Partially obstructed	Unobstructed	Visible center
Case 1	6	3	15	18
Case 2	0	4	20	24
Case 3	10	1	13	14
Case 4	3	3	16	19

## 4 Discussion

The experimental results have demonstrated the preliminary feasibility of our robotic solution for diagnosing and monitoring COVID-19 patients’ lungs, by successfully imaging specified lung infiltrates in an autonomous mode. In a real-life setting, a sonographer initially palpates the patient’s torso to assess the location of the ribs for correctly positioning the US probe. We proposed a solution that autonomously replicates this behavior, by collecting force–feedback displacement data in real time. This approach has been assessed in simulation on three patients with varying BMI. We have evaluated our algorithm for proposing optimal US probe scanning positions and orientations given a patient’s CT scan for generating target points for the robotic system. We also addressed the issue of potentially unavailable prior CT scans by developing a deep learning model for ribcage landmarks estimation. Our approach has demonstrated feasibility potential in both simulations and physical implementation, with a significant improvement in US image quality when using force feedback, and comparable results to CT experiments when using ribcage landmarks prediction only. The successful imaging of the target points in simulation, as well as the reduction in shadowing effects in the phantom experiments, is deemed to fulfill the clinical requirements defined in Subsection 3.3. This does not, however, imply the system’s clinical feasibility, as additional experiments would be needed to support such claim. The current system is intended to alleviate the burden of continuously needing highly skilled and experienced sonographers, as well as to provide a consistent solution to lung imaging that can complement a clinician’s expertise, rather than eliminating the need of a clinician’s presence.

We acknowledge the limitations associated with the current implementation. The presented work is a study for demonstrating the potential of the methods’ practical feasibility, laying down the foundation for more advanced experimental research. Although the simulated environment has been carefully chosen in an attempt to replicate real-life settings, and the physical phantom results have shown to support the simulated results, further experimentation is required to validate the outcomes. The simulated environment is incapable of capturing all the nuances of a physical setup—soft tissue displacements, for instance, have been acquired from a limited set of points on the patients’ torsos using finite element simulations, with displacements in other locations obtained by interpolation. Elements such as breathing and movement of a patient during the scanning procedure are additional, albeit not comprehensive, factors too complex to be modeled in a robotic simulation, but ones that can negatively affect the outcome of the implementation.

Future work will focus on developing improved registration techniques to reduce the error associated with this step. An image analysis component as an additional feedback tool can further enhance the accuracy of the setup; force feedback ensures that the US probe is not positioned on ribs, and that it is in contact with the patient; however, it does not guarantee image quality. Additionally, a larger sample size of patients needs to be investigated to better validate the proposed force–displacement hypothesis. As was shown in Figure 4, different patients showed different displacement magnitudes, with the least noticeable differences pertaining to the patient with the highest BMI. It is important to understand in the future how well this approach scales with different body types and genders. The physical phantom used for the experimental setup does not contain lungs, has Polycarbonate bones, and embedded hemorrhages made of water balloons. These factors make the US image analysis quite challenging, with room for misinterpretation. Additional experiments need to be conducted on phantoms containing structures that can be clearly visualized and distinguished in US to better assess the experiments’ outcome. Lastly, this study does not account for variations in patients’ posture as compared to their posture when the CT scan was taken. The combination of CT data, ribcage landmark estimation, and force feedback will be investigated in future work for improved imaging outcomes.

## 5 Conclusion

We have developed and tested an autonomous robotic system that targets the monitoring of COVID-19-induced pulmonary diseases in patients. To this end, we developed an algorithm that can estimate the optimal position and orientation of an US probe on a patient’s body to image target points in lungs using prior patient CT scans. The algorithm inherently makes use of the CT scan to assess the location of ribs, which should be avoided in US scans. In the case where CT data are not available, we developed a deep learning algorithm that can predict the 3D landmark positions of a ribcage given a torso surface model that can be obtained using a depth camera. These landmarks are subsequently used to define target points on the patient’s body. The target points are relayed to a robotic system. A force–displacement profile collection methodology enables the system to subsequently correct the US probe positioning on the phantom to avoid rib obstruction. The setup was successfully tested in a simulated environment, as well as on a custom-made patient-specific phantom. The results have suggested that the force feedback enabled the robot to avoid skeletal obstruction, thus improving imaging outcomes, and that landmark estimation of the ribcage is a viable alternative to prior CT data.

## Data Availability

The raw data supporting the conclusions of this article will be made available by the authors, without undue reservation.
